# A low-cost, novel endoscopic repeated-access port for small animal research

**DOI:** 10.1016/j.mex.2020.101049

**Published:** 2020-08-29

**Authors:** Shannon N. Ingram, Andrew B. Robbins, Stacy J. Gillenwater, Vince Gresham, James C. Sacchettini, Michael R. Moreno

**Affiliations:** a Department of Biomedical Engineering, at Texas A&M University, United States; b Department of Mechanical Engineering, at Texas A&M University, United States; c Department of Poultry Science, at Texas A&M University, United States; d College of Veterinary Medicine, at Texas A&M University, United States; e Department of Biochemistry and Biophysics, at Texas A&M University, United States

**Keywords:** Endoscopy, Manufacturing, Molding, Silicone, Implantable, Device

## Abstract

Repeated endoscopic access to the abdominal cavity of animal models is useful for a variety of research applications. However, repeated surgical access may affect the welfare of the animal and compromise results. We present the design and benchtop manufacturing process for a self-sealing endoscopic port requiring surgical incision only at implantation. It can be used for repeated body cavity access over a long time period. This device reduces costs, animals required for a given study, and potential suffering for each animal. This novel endoscopic port is designed for low-cost benchtop manufacturing without expensive equipment such as injection molding facilities. Devices manufactured using the method described in this work have been implanted successfully in hen models for investigation of ovarian cancer for over two years. All work followed Texas A&M University institutional guidelines and was covered under Animal Use Protocol 2017–0172, approved by TAMU Animal Care and Use Committee (IACUC).

This method can be translated to produce similar devices for use in other small animal models besides the galline model used in this work.

This method can be used to produce devices for slightly different purposes than repeated endoscopic access, such as production of an entry port for surgical tools.

## Graphical abstract

**Figure:**
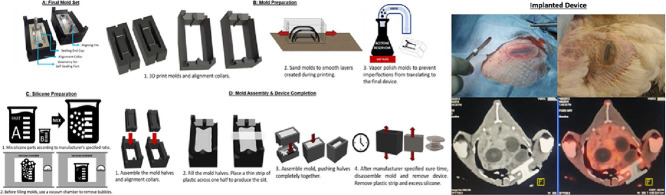

Specifications table

**Table:** 

Subject Area	Veterinary Science and Veterinary Medicine
More specific subject area	Repeated endoscopic evaluation of small animal disease models
Method name	Low-Cost Manufacture of an Endoscopic Repeated-Access Port for Small Animals
Name and reference of original method	N/A – This method was developed because there were no available solutions to allow veterinarians studying ovarian cancer to access interior cavities of avian disease models without making repeated incisions over time.

## Method details

### Background

Repeated endoscopic access to the abdominal cavity of animal models is useful in a variety of research applications, including observation of pathology development in this area of the body [8]. Surgically accessing body cavities in a minimally invasive manner may reduce scarring and recovery times. In research applications, an endoscopic port may be created by surgically modifying and rearranging tissue [5], though typically endoscopic procedures are performed through a cannula that provides direct access into a vein or body cavity. However, because cannula insertion is often the highest risk part of the endoscopic procedure, increased pain and risk of infection for the patient make repeated entry into the same body cavity over long periods of time impractical [3].

Cannulas have been designed to optimize one-time laparoscopic operations involving use of a channel to traverse the wall of a body cavity [4]. For example, use of a magnetic anchoring system reduces scarring and other complications [2]. Peiro, et al. developed a single-access fetal endoscopy method and studied its potential use in the investigation of myelomeningocele in sheep models [6]. Cannulas with sealing properties that maintain pressure differentials while surgical tools are in use have also been developed. These include (1) an elastic sealing ring that simplifies laparoscopic surgery and provides multiple degrees of freedom for surgical instruments, (2) an access port with a flexible, sealing cannula that gives more surgical freedom, and (3) a self-sealing cannula cap allowing resealing before and after removal of a surgical instrument [1,7].

While these devices may improve patient outcomes, they are not appropriate for long-term applications that require repeated non-surgical access, e.g., tracking disease development or effectiveness of a therapeutic intervention in an animal model. Devices designed for repeated access in long term applications include the rumen cannula, which is used to access the rumen in ruminants. These devices can remain implanted for months or years at a time. Such devices have also been used in pigs to provide access to the digestive tract [9]. However, without major modification, a rumen cannula is not appropriate to provide access to interior body cavities due to of the risk of infection in those areas. In human medicine, implantable port devices are used regularly to provide long term access to blood vessels; however, these are unsuitable for use in accessing larger body cavities that require larger laparoscopic instruments.

Currently, there is no device that permits repeated endoscopic access to the body cavities of animal models. Instead, a new incision must be made for each procedure, increasing the animal's stress, pain, and risk of surgical complications, including infection. The primary objective of this work is to present a novel self-sealing endoscopic access port to address this need and to describe in detail its design and manufacturing process to enable its reproduction by other researchers. The device has been designed for benchtop manufacturing (via three dimensional [3D] printing) at a low cost without expensive equipment like injection molding facilities. We have successfully used the device in a galline model of ovarian cancer, eliminating repeated surgical procedures that would have been required to visually track the cancer's development. The outcomes of the use of the device within the context of the ovarian cancer study are briefly summarized (including animal response to implantation and resulting changes in the device design) to demonstrate its feasibility and promise for use in future studies.

The ease of manufacture, deployment, and use of this device suggest that it can easily be adapted to a variety of animal models to improve animal welfare by reducing the number of animals necessary (by preventing loss due to surgical complications) and refining testing procedures to minimize suffering (by decreasing surgeries per animal and resulting pain and distress) while simultaneously increasing time for viable data collection.

### Materials and methods

**Fig. 1:**
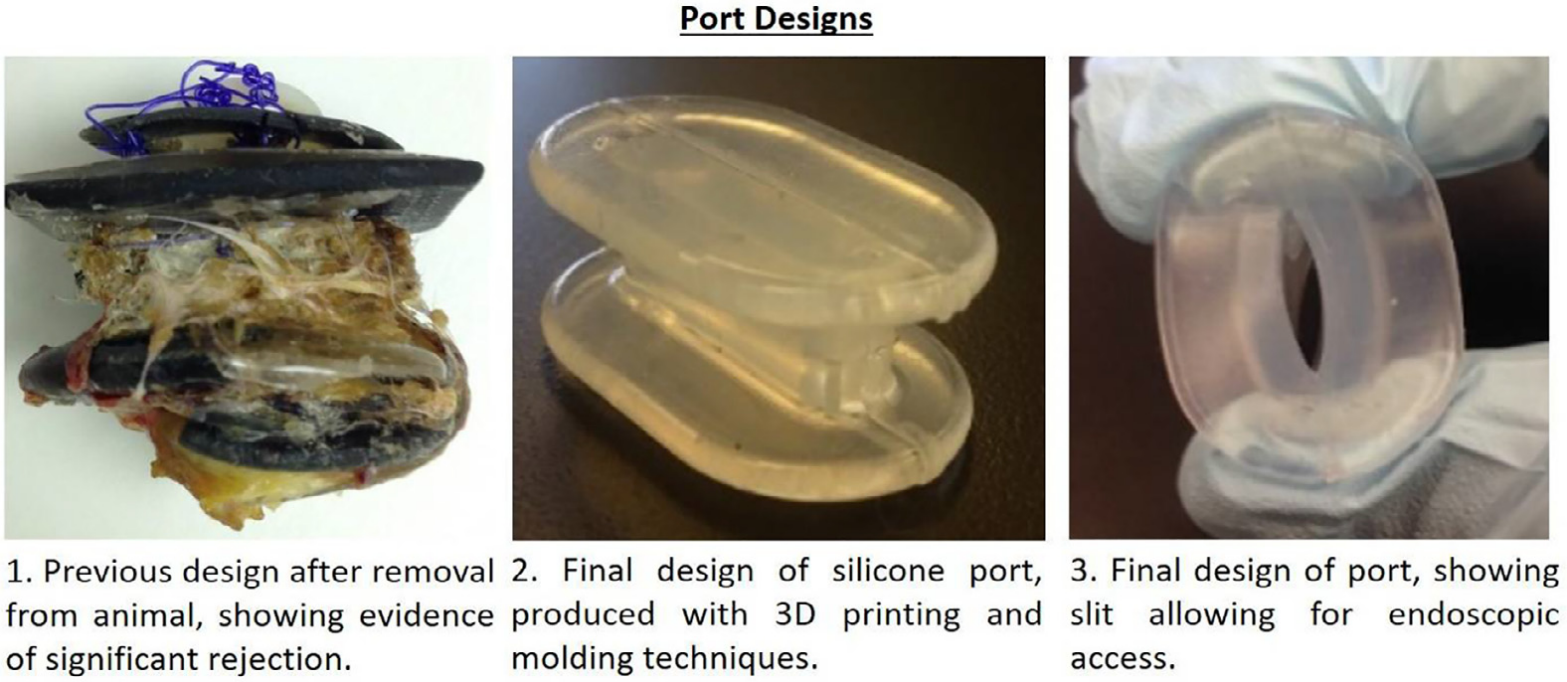
Evolution of Cannula Designs. LEFT: An initial port design made of 3D printed material, using a twist cap to allow access into the body cavity. Removed from animal model after rejection. MIDDLE: A completed cannula of the final design. RIGHT: Final design in open configuration.

In converging to the present incarnation, numerous design iterations were produced and tested. We will not describe in detail all of these iterations, but some discussion is warranted to highlight important design considerations that emerged in the process. The first iteration that was implanted was a rigid ring 3D printed out of polylactic acid (PLA) plastic using a MakerBot Replicator 3D printer (Makerbot Industries, New York, NY). This device had several shortcomings: first, the shape of the rigid device allowed only limited endoscopic movement; second, the device's rigidity complicated implantation; and third, although the material itself was biocompatible, the animals still rejected the device, likely due to its geometry and rigidity. Another iteration of the device design that performed well on the benchtop and was subsequently implanted, was 3D printed in two materials (a rigid material for the central core, and a rubber-like material for the inner and outer surfaces) with a Connex 500 PolyJet 3D printer (Stratasys, Ltd., Eden Prairie, MN) (Fig. 1, Left).

This design also contained a removable plug. The 3D printed materials are not biocompatible, so a number of encapsulation methods (silicone, nitrocellulose) were attempted to produce a barrier between the 3D printed material and the tissue of the animal. These attempts were not successful, and this design was abandoned. Fig. 1 (Left) shows the explanted device with buildup of granulation tissue indicating rejection by the animal model. The animal implanted with this device was euthanized, and the explanted device was evaluated. The final design of the self-sealing endoscopy port (Fig. 1, Middle) is composed of two self-sealing end caps separated by a self-collapsing tube made of medical grade silicone (Fig. 1, Right). This design differed from previous attempts; it was a single piece and a single, biocompatible material. The port is implanted so one sealing end cap is against the skin of the animal and the other is inside the body cavity. The self-sealing port then allows an endoscope to pass from outside the body into the internal body cavity without requiring repeated surgical incisions, and the port self-seals when the instrument is removed.

**Fig. 2:**
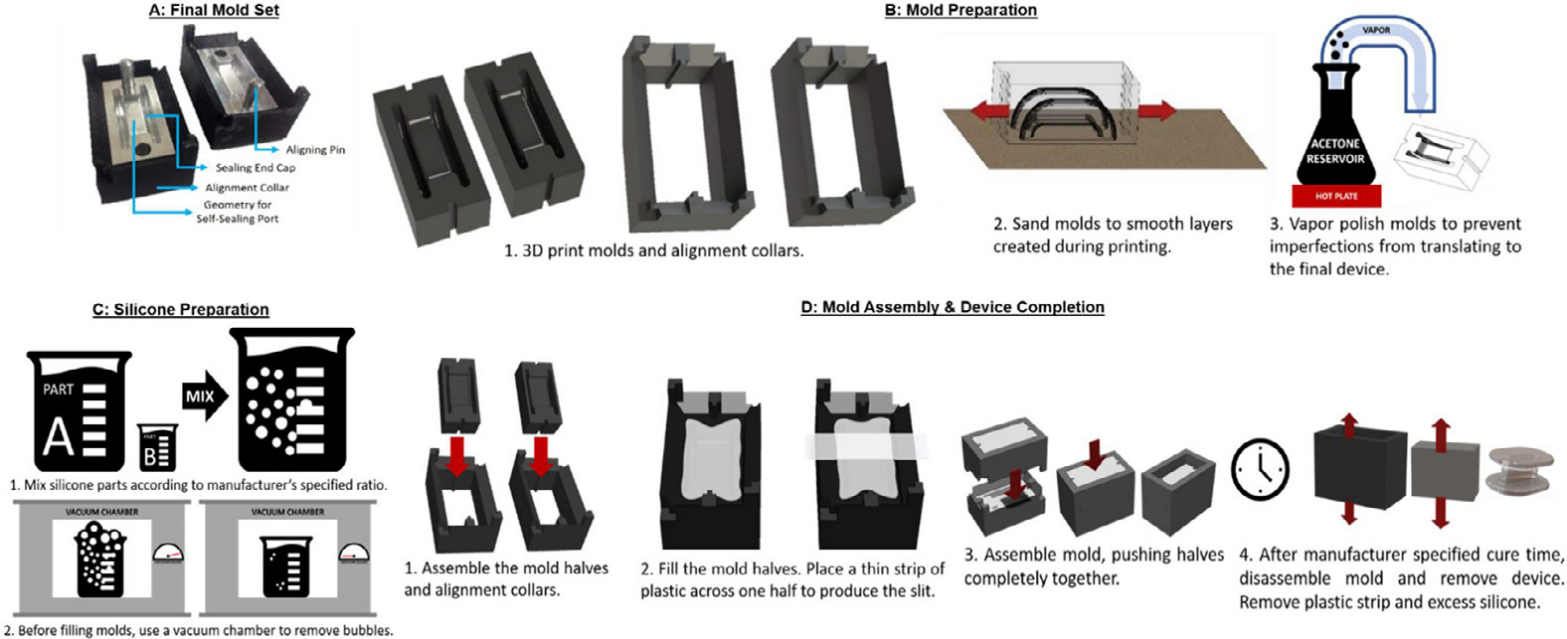
Manufacturing Process for Devices. A) Final Mold and Alignment Collars. The molds were designed to produce the precise curves required for the sealing end caps and self-sealing port. Alignment pins and collars were designed with interlocking geometries to ensure the mold halves could be consistently assembled correctly. B) Mold Preparation Process. TOP: Mold and collar design. BOTTOM LEFT: Sanding of mold. BOTTOM RIGHT: Vapor polishing of mold. C) Silicone Preparation. TOP: Mixing of silicone. BOTTOM: Degassing of silicone in vacuum chamber. D) Mold Filling & Device Completion. TOP LEFT: Assembly of 3D printed parts. TOP RIGHT: Mold filling and plastic strip placement. BOTTOM LEFT: Mold closure. BOTTOM RIGHT: Mold opening and device clean up after cure time.

Once this design was selected, a small-batch benchtop manufacturing process was developed. A two-part mold with alignment collars was designed using the university licensed version of Solidworks (Dassault Systèmes SE, Vélizy-Villacoublay, France). These molds were 3D printed using acrylonitrile butadiene styrene (ABS) filament on a Makerbot Replicator 2X. The 3D printed molds were used during design development to reduce prototyping time and costs, but eventually aluminum molds were produced to increase durability (Fig. 2A).

Due to the extrusion-based manufacturing process, the surfaces of the 3D printed molds used during design development were not smooth. To address this, the molds were sanded (Fig. 2B) and vapor polished with acetone (Fig. 2B). Initial sanding was performed with coarse grit sandpaper, working to finer grit sizes later in the process. Small files were used to access the parts of the mold that were more difficult to reach. Alternatively, sand blasting techniques could be used.

After the molds were sanded, they were vapor polished with acetone to produce a glossy finish. Briefly, acetone was boiled in a glass reservoir. The vapor was allowed to escape through a chemically resistant hose and directed toward the component being polished. The hose was held so the vapors could be “poured” onto the mold, dissolving the surface layer of the ABS plastic and allowing it to spread evenly on the surface of the mold.

An important consideration when vapor polishing ABS is that pinholes could be created in the surface of the print during the process. Because the molds are mostly hollow, holes that allow air to escape from the mold could cause bubbles when the molds are filled. Another concern is the effect of excessive acetone vapor on ABS; the plastic could become brittle and crack, also leading to bubbles in the mold. Care should be taken to minimize excess vapor polishing, and test pieces should be polished before production pieces are attempted. Vapor polished molds should rest for at least 48 h, allowing remaining acetone to evaporate. Otherwise, bubbles may form in the plastic or the molded component as the remaining acetone outgasses. Alternatively, commercial vapor polishing systems could be used, but the method presented herein requires minimal specialized equipment.

Once the molds are smooth, they can be filled. For this study, silicone elastomer acquired from Applied Silicone (LSR 40 – 10:1 A:B, Implant Grade, Part #40,082) was used. Applied Silicone was purchased by NuSil Technology, LLC (Carpinteria, CA), and an identical product can be purchased through that company (Products MED-4244, MED0–4244). The silicone product is a two part, medical grade, platinum catalyzed silicone elastomer system. Combination of the Part A and Part B components crosslinks the product to form a vulcanized, medium durometer, high strength silicone rubber, making the devices very durable.

Silicone was prepared according to manufacturer specifications before distribution into the cannula molds (Fig. 2C). The mixing container should be approximately 10 times the volume of the silicone prepared to allow expansion of the material during the degassing process, described later. Part A was poured into the container and weighed, and 10 percent of that weight of Part B was added (according to the manufacturer's recommended ratio). The parts were mixed together; production of air bubbles in this process is unavoidable but should be minimized (Fig. 2C).

To reduce air bubbles, the material was degassed in a vacuum chamber at room temperature (Fig. 2C). A vacuum pump was used to bring the pressure in the chamber to about 29 inHg vacuum and held for 3 to 5 min before release. This process was repeated until most bubbles in the silicone had deflated, although consideration should be given to the manufacturer specified working time of the silicone product to avoid curing during degassing.

After the silicone was prepared, the molds and alignment collars could be assembled, filled, and set to cure (Fig. 2D).

The molds were inserted into the alignment collars (Fig. 2D). The silicone was poured into the molds, overfilling each mold (Fig. 2D) to ensure complete filling. Each half of the mold was then degassed again according to the procedure described previously.

A polyethylene sheet (width: 20 mm, thickness: 0.254 mm) was placed across one half of the mold to create the slit in the device (Fig. 2D). The alignment collars were interlocked, and the molds were pushed together (Fig. 2D). The filled, closed molds were warmed at 66 °Celsius for 2–3 h, increasing time as necessary when more molds were being used (Note: for some silicone materials, heated curing may not be necessary or beneficial – refer to manufacturer's instructions).

The molds were taken out of the oven, removed from their collars, and split apart, exposing the silicone port (Fig. 2D). Excess silicone was cut and the plastic strip was removed from the slit. The port was ready for sterilization (in this case, through chlorhexidine soak followed by sterile saline or gas sterilization by ethylene oxide) and implantation.

3D printed molds were adequate for manufacturing ports during design and prototyping and could have undergone continued use. However, once a final design was chosen, more durable aluminum molds were produced using computer aided manufacturing. The Solidworks part files were converted into parasolid binary files and manufactured using a Computer Numerical Control (CNC) machine to reduce cost and time.

### Method validation

A self-sealing endoscopy port was produced to allow easy visual access to body cavities. After several design iterations that showed signs of rejection after implantation in hen models, a design was developed that has been successfully used in hens with implantation times of more than two years (AUP# 2017–0172). This precise geometry was achieved by repeated production of high quality iterations of the port for evaluation and test implantation by collaborators, who could then give feedback to further perfect the design. The device was designed to reduced scarring and infection, also aided by the self-sealing characteristic and the biocompatibility of the silicone used.

A low cost, small-batch benchtop manufacturing process has been developed and described to allow easy production of this device and potential adaptation for other purposes. Devices produced with this method were then used in hens to study the development of ovarian cancer while eliminating the need for repeated surgeries and preventing infection or further injury to the animals.

During implantation, the animals were anesthetized using isoflurane induction (5%) and maintenance (2.5–3%). Feathers were removed and the skin was cleaned with betadine and alcohol. A 1.5 – 2.5 cm incision was made on the left side of the body wall, caudal to the last rib in order to access the active ovary in the hen. A sterilized (autoclaved) port was placed into the incision and sutured to the body wall using 2–0 polydioxanone suture (PDS). The animals were recovered by removing anesthesia and administering 1 mg/kg buprenorphine IM, followed by 3 mg/kg carprofen to provide pain relief. After 12 h, the animals were evaluated for pain – veterinarians watched for limping, lack of weight bearing or rousing, holding wing down or leg up, lack of defecation, and lethargy. The animals consistently recovered from surgery rapidly, commonly laying eggs following or even during the surgery.

**Fig. 3:**
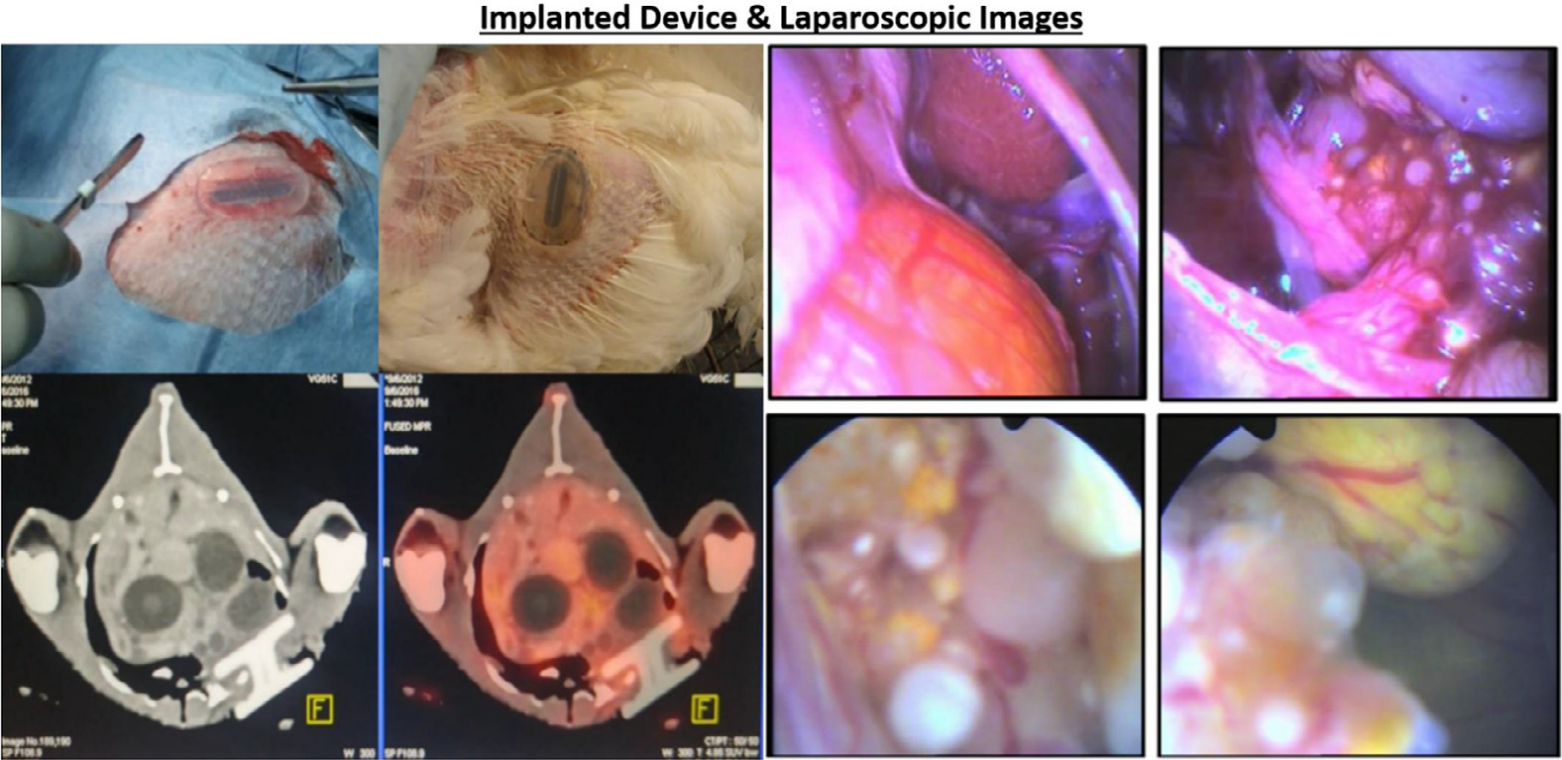
Implanted Device. TOP LEFT: Initially implanted cannula. BOTTOM LEFT: Computed Tomography (CT) and Positron emitted tomography (PET) image overlay. TOP RIGHT: Laparoscopic images of healthy tissue. BOTTOM, RIGHT: Laparoscopic images of cancerous tissue.

The final design, shown in Fig. 3, allowed successful, repeated access to hens’ ovaries for laparoscopic visualization and biopsy with a 4 mm rigid laparoscope (Portoscope.com, Inc) lubricated with sterile ointment, as well as chemotherapeutic delivery (after the same anesthesia process that was used for implantation) without the use of capnoperitoneum. This procedure required only one person to restrain the specimen briefly (<1 min) for anesthesia (identical to what was given prior to implantation), then to perform the laparoscopy. The data produced was not directly compared to images from a camera, but the veterinarians using the images were pleased with their quality.

Based on the external appearance of the implant site and observation of the hens’ behavior in comparison with hens who did not undergo device implantation, the veterinarians and veterinary technicians involved in the study saw no signs of rejection, scarring, infection, or other problems. Additionally, after one year of port placement, a board certified veterinary pathologist performed necropsies on a subset of the animals to observe the tissue surrounding the port. The tissue around the port was healthy, and the port was intact and maintained a seal with the skin. Due to the low number of animal subjects and the qualitative nature of the data acquired, no statistical analyses were performed, but the device has been used successfully in hens with implantation times of more than two years, as shown in Fig. 3 (1st row, 2nd column). All six hens implanted with the final device design continued through the primary study (i.e. were not euthanized or removed from the study due to negative effects of implantation).

It should be noted that the design of this port can be altered depending on the animal model and specific body cavity it might be used in. These parameters may warrant changes in basic dimensions (height, width, thickness, seal length, etc.). Changes in material may also be warranted to help adapt to dimensional changes, such as using a more compliant silicone to overcome reduced size that limits the endoscopic tools that may be used. Changes in these parameters would directly impact the maximum diameter and number of usable instruments, as well as the interventions that may be possible for a given study. However, while some design iteration may be required, we believe that there are a wide range of animal models and research contexts this molding method may be used in to allow more efficient use of study animals.

## Declaration of Competing Interest

The authors declare that they have no known competing financial interests or personal relationships that could have appeared to influence the work reported in this paper.
